# Can AI find the cavities in caries prediction and diagnosis?

**DOI:** 10.1038/s41432-025-01181-0

**Published:** 2025-07-26

**Authors:** Lucy Bennett, Lucy Tiplady, Greig Taylor

**Affiliations:** 1 Dental Core Trainee, Newcastle Dental Hospital, Newcastle upon Tyne, UK; 2 Newcastle University, School of Dental Sciences, Newcastle upon Tyne, UK

**Keywords:** Paediatric dentistry, Dental caries, Preventive dentistry

## Abstract

**A Commentary on:**

**Rokhshad R, Banakar M, Shobeiri, P, Zhang P**.

Artificial intelligence in early childhood caries detection and prediction: a systematic review and meta-analysis. *Pediatr Dent*. 2024;46:385–394.

**Data sources:**

A literature search was performed in May 2024 via PubMed, Scopus, Embase, Web of Science, Institute of Electrical and Electronics Engineer database sources, and across the grey literature. Further studies were identified after analysis of reference lists. The research question was defined using the population-intervention-comparison-outcome (PICO) framework.

**Study selection:**

Studies published between 2010 and 2024 were included, that used artificial intelligence (AI) algorithms including machine learning (ML), deep learning (DL) and neutral networks (NN) for detecting and predicting early childhood caries (ECC). Exclusion occurred where the full text was inaccessible and non-English papers. Two independent reviewers screened titles and abstracts, with the use of a third reviewer in the case of any disagreement. The process was then repeated with the full texts to assess eligibility, again with a third reviewer where necessary. A total of 21 studies were used in the final analysis following assessment, 7 of which described ECC detection, and 14 for ECC prediction.

**Data extraction and synthesis:**

The extracted data included author, publication year, study objectives, data modalities, datasets, annotation procedures, follow ups, ML test, AI model architecture, outcome measures and evaluation metrics. The findings were summarised descriptively. Quantitative synthesis was performed on six studies that reported sensitivity and specificity. Summary receiver operator characteristic curves were used to assess discriminatory ability. Statistical analysis was completed.

**Results:**

A total of 21 studies were included in the final analysis. It revealed that AI based methods, especially DL algorithms showed promising results in detecting ECC, with accuracy range of 78–86%, sensitivity of 67–96%, and specificity from 81–99%. ECC prediction had accuracy range of 60-100%, sensitivity of 20–100%, and specificity of 54–94%. The pooled sensitivity and specificity of these studies was 80% and 81% respectively, with confidence intervals of 95%, indicating statistically significant effects.

**Conclusions:**

AI has demonstrated substantial potential in the detection and prediction of ECC. Further research is required to refine the technology and establish its application in paediatric dentistry.

## GRADE Rating:



## Commentary

Early childhood caries (ECC) is defined as ‘the presence of one or more decayed (non-cavitated or cavitated lesions), missing (due to caries), or filled tooth surfaces in any primary tooth in a child 71 months of age or younger^1^. A global estimated pooled ECC prevalence of 49% has recently been reported^2^. The impact of caries in the primary dentition includes pain, infection, missed school, financial cost for the family, and significant burden on health services. Prevention is key, and the use of AI may be able to assist in identifying and predicting caries, allowing us to target our efforts and reduce risk factors.

Systematic review and meta-analysis offer the highest level of evidence, allowing a robust assessment of the current available evidence and conclusions to be drawn based upon a strong statistical basis. Six studies were included, providing a limited amount of data for analysis. Despite being appropriate, the inclusion criteria may have been too strict as ongoing studies and non-English studies were excluded. However, more realistically, the limited studies suggest a paucity of data in this field given how novel the use of AI is in the dental field. The pooled data was presented clearly; however, the reported high level of heterogeneity are likely to reduce the external validity of this paper.

The review addressed its limitations, including AI development in isolation from clinical settings, and how performance may vary in practical application^3^. The paper discussed the limitation of small sample sizes, which may affect the generalisability of the findings. Additionally, studies may have been limited to a restricted cohort, these may not fully translate to the wider paediatric population. The authors highlight the need for further research including larger scale validation. The study also discusses the significant issues of lack of standardisation across the different AI methodologies (ML, DL, neural networks). As discussed, pooling the data did not aid analysis. The lack of uniformity can make it difficult to compare results across studies and draw consistent conclusions. Recognition of this inconsistency leads the authors to recommend set guidelines for reporting metrics in studies involving AI.

The article suggests the use of AI in ECC detection could be beneficial in comparison to traditional diagnostic methods by clinicians alone. However, the current pooled estimates, CI and the range of sensitivity and specificity, suggest otherwise. Due to this, AI could currently compliment clinician acumen, acting as an additional investigation for greater detection of ECC and conferring with technology would allow us to achieve a potentially greater accurate diagnosis. However, the ultimate aim would be to surpass ECC detection of experienced clinicians, reducing human error. The authors identify this could help achieve more efficient and accessible practice; however this appears a long way off.

Advanced ECC detection and prediction methods could allow for a more informed approach to patient care, assisting clinicians in tailored prevention and AI tools have the potential to provide this. Detection tools could be particularly useful in less experienced practitioners. Generation of caries risk level from AI aided screening could revolutionise the way we implement guidelines into our practice. Utilising AI for caries detection and risk assessment could allow the clinician to offer a more personalised approach to care including patient profiles, diet and oral hygiene regime.

In summary, the use of AI has the potential to advance paediatric dentistry by decreasing the burden of untreated primary caries. Alongside the evidence highlighted, further research should be undertaken into how to apply AI technology into clinical situations with further long-term studies to solidify the evidence base.

Practice Points

- AI shows promising ability to detect and predict ECC.
- AI can be used to identify caries risk and allow tailored prevention plans.
- Further research should be conducted with longer term follow ups and with standardised approaches to provide more consistent and accurate evidence.
